# Ion mobility and material transport on KBr in air as a function of the relative humidity

**DOI:** 10.3762/bjnano.10.203

**Published:** 2019-10-30

**Authors:** Dominik J Kirpal, Korbinian Pürckhauer, Alfred J Weymouth, Franz J Giessibl

**Affiliations:** 1 Institute of Experimental and Applied Physics, University of Regensburg, 93040 Regensburg, Germany

**Keywords:** ambient conditions, atomic force microscopy, material transport, relative humidity

## Abstract

Surfaces exposed to air can change their structure due to external influences such as chemical reactions or material exchange and movement. The adsorbed water layer that is present under ambient conditions plays an important role especially for highly soluble materials. Surface atoms can easily diffuse into the thin water layer and, when surface conditions are favorable, they can re-attach to the surface. We collected atomic force microscopy images of KBr surfaces in a humidity-controlled glove box at various relative humidities below 40%. By scratching and poking the surface with the AFM tip, we constructed energetically unfavorable holes or scratch sites and material accumulations and recorded the evolution of these defects as a function of the time. We observed an exponential decay of the size of the defects and material accumulations, and from this data we determined energy barriers to dissolution and aggregation of approximately 0.9 eV.

## Introduction

Defining surface properties under ambient conditions is challenging as they are heavily influenced by the environment. In general, there are various contributing factors such as temperature, air pressure and air composition. Typically, air is composed of different gases, a small fraction of aerosols and water vapor. The relative humidity (RH) usually ranges from about 25% to 70%, depending on weather and season. A thin film of water molecules adsorbs on every surface exposed to humid gases [1–3].

The thickness of these water layers depends on many factors including the relative humidity, surface roughness, hydrophilicity or hydrophobicity, meniscus formation (as described later) and also air pressure and temperature [2]. In the case of freshly cleaved or dried surfaces the amount of adsorbed water also relates to the time of exposure to the humid air. The layer thickness ranges from partial coverage at very low humidities (RH *<* 10%) up to several nanometers near saturation. Arima et al. performed XPS measurements on potassium bromide (KBr) thin films under UHV conditions (4·10^−10^ mbar) in order to determine the thickness as a function of the relative humidity [4]. They observed an increase of the coverage up to one monolayer at RH = 30%. This thickness remained constant up to a relative humidity of about 60%. A small increase up to RH = 80% and large increase up to 2 nm for higher humidities was recorded. It has to be taken into account that measurements at such low pressure rather relate to the outer atmosphere than to ambient conditions. However similar observations have been made by Asay et al. under ambient conditions, who showed on silicon oxide the growth of three monolayers up to RH = 30%, an additional layer forming by increasing RH to 60% and further growth of the water film up to 2.7 nm (ca. 10 monolayers) thickness at higher humidities [1].

On the atomic level, water and adsorbed molecules can arrange according to the surface structure and form ordered hydration layers that are also referred to “ice-like” [1,5–8]. The presence of water can have a large influence on the surface, especially for salt crystals. Investigations suggest that the presence of water and, as a consequence, the relative humidity have a direct influence on material transport and step movement [3,7]. The relationship between humidity, water coverage and movement speed, however, is complex.

In this study we investigated the surface of KBr, a salt crystal, by using frequency-modulation atomic force microscopy (FM-AFM) using a qPlus sensor [9–11]. The aim of our experiments is a qualitative and quantitative observation of the change and evolution of the KBr surface as a function of the relative humidity. Therefore, several artificial defects in the range of some to tens of nanometers were created and observed over a period of a few hours up to a few weeks while the relative humidity was kept within a certain range for each experiment.

## Experimental

For the experiments we used a custom-designed AFM equipped with a qPlus sensor. The qPlus sensor is a stiff (*k* = 1800 N/m) self-sensing quartz sensor with a resonance frequency around *f*_0_ = 32 kHz. It has enabled unprecedented results in low-temperature AFM, such as the imaging of single pentacene molecules by Gross et al. [12], intramolecular resolution of PTCDA at room temperature by Huber et al. [13], as well as the capability to perform non-destructive measurements on sensitive biological samples in air and in a liquid [14]. Moreover, it has been shown that a qPlus AFM is capable of observing material dissolution [7,15].

These studies show that the high stiffness is beneficial and allows one to use larger tips built from any appropriate tip material and to operate the AFM at small amplitudes without risking jump to contact [7,16–19]. A small and sharp splinter of a smashed sapphire bulk crystal was used as a tip. Sapphire is a very hard material, it is hydrophobic with a contact angle to water above 80°[20], and it is chemically inert [21]. The high hardness allows us to create large artificial defects in our sample (as described later) without damaging the tip. Moreover, the hydrophobicity reduces capillary formation at the tip–sample contact area. Capillary condensation causes a locally enhanced presence of condensed water molecules [22], which might correspond to a much higher relative humidity near the apex. Furthermore, the water film and the water meniscus that forms around the tip–sample contact cause damping forces on the probe, which result in higher noise [7]. The hydrophobicity of sapphire reduces the meniscus effect. The impact that hydrophobicity or hydrophilicity of the tip material can have is demonstrated by Wastl et al. in [23], by comparing the rip-off distance of the meniscus for a sapphire and a silicon tip.

In order to observe a significant temporal evolution of the sample surface we chose KBr, a soft and easily soluble salt (650 g/L in H_2_O at 20 °C) [24]. KBr crystallizes in the rock-salt structure with a lattice constant of *a* = 660 pm [25]. A clean and dry surface can be generated by cleaving the bulk crystal with a knife along the [100] direction. The surface shows large atomically flat terraces with heights ranging from one to a few atom layers. Steps, screw dislocations, small holes and islands can be observed. However, it was not possible to observe single atomic defects, as their life time is too short for the AFM imaging process [7].

The soft sample material allows us to generate large and deep artificial defects on the scale of some tens of nanometers in length and some nanometers in depth. We deliberately created two types of defects: poking holes and scratching holes. Poking holes are deep and narrow holes, created by poking the tip several nanometers into the surface. This results in a steep hole with a shape similar to the tip apex. The removed material mainly accumulates around the hole but partially attaches to the tip. Scratching holes are larger defects, created by poking the tip into the surface and then scanning quadratic or rectangular areas with a freely determined edge length, of typically some tens of nanometers. The result is an almost rectangular hole with the removed material accumulated around the defect and to a small extent attached to the tip. Most of the material accumulates at the turnaround points of the tip. The scratching technique creates larger and more reproducible holes than the poking of single points, since the shape of the tip becomes less important.

**Figure 1:**
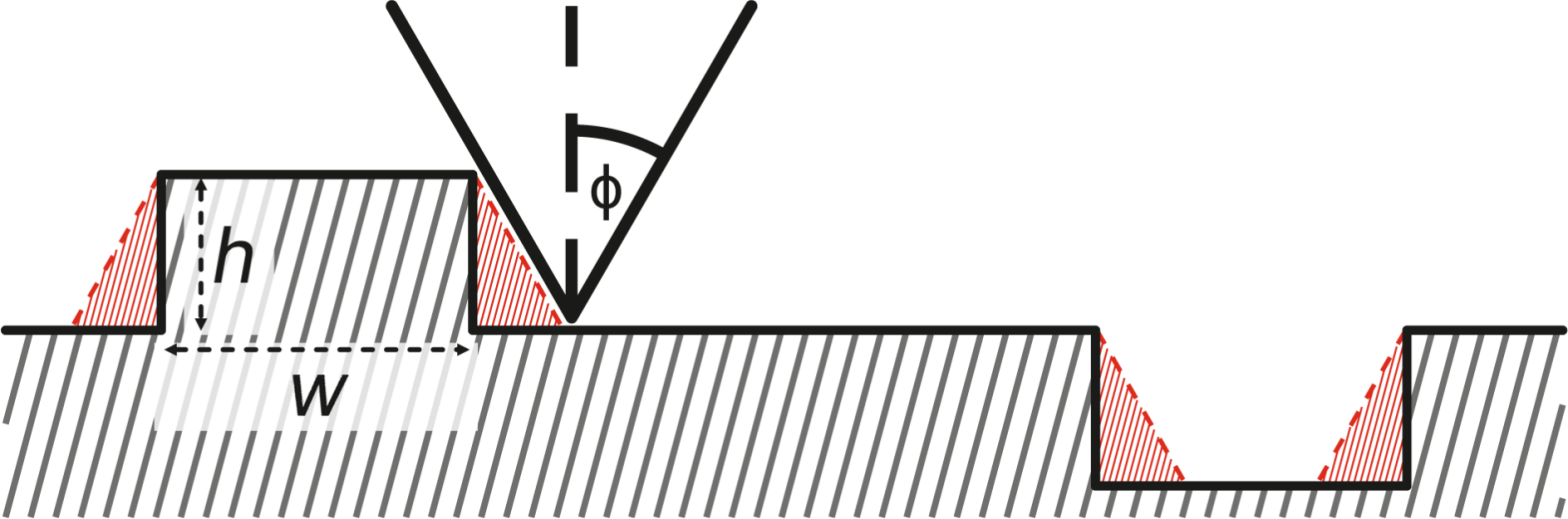
1D schematic of the tip–sample convolution at islands and holes. Due to the tip geometry step edges appear smeared out. Consequently, accumulations appear to be larger and holes smaller. The red hatched areas show the misassigned volumes at step edges. This effect is more pronounced for large tip angles (blunt tips) compared to small angles (sharp tips).

AFM enables high lateral resolution, although the imaging of structures with rough topographies is challenging. When scanning rugged surfaces the back structure of the tip apex becomes more important. As the tip follows the surface while keeping the tip–sample interaction constant, side effects of the tip become more important for steep edges. The depicted structures show a convolution between the tip and sample. This may result in smeared out step edges in the images and, consequently, an amplification of convex structures such as material accumulations, as well as a reduction of concave structures [26–27] such as holes as shown in Figure 1. These artifacts can be minimized by using sharp tips with small tip angles ϕ, as defined in Figure 1. If we consider the apparent size of a surface feature with width *w* and height *h*, for a two-dimensional case, the measured cross-sectional area *A* of the elevation (+) or hole (−) is given by:

[1]
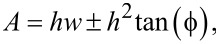


and the measured volume *V* for circular structures having the real volume of π*h*(*w*/2)^2^ is given by:

[2] $$V=\pi h\left(\frac{w^{2}}{4}\pm \frac{wh}{2}\text{tan}\left(\phi \right)+\frac{h^{2}}{3}\tan^{2}(\phi )\right).$$

Thus, even if only the material from a hole ends up forming an elevation, the measured volume of the elevation is apparently larger than the volume of the hole.

The relative humidity in laboratories without air conditioning usually ranges from 25 to 60%. In order to provide a stable relative humidity for time periods up to some weeks, the microscope was placed into a glove-box. The humidity inside the box can be increased by evaporating water or decreased by using the drying effect of silica gel. With this setup, RH values below 2% can be obtained. The desired humidity can be reached within a time scale ranging from some minutes up to a few hours. Without active influence the air humidity remains quite constant with a maximum rate of change of less than one percent per hour. The humidity was continuously measured and, if needed, adjusted during the measurement process.

All AFM experiments were performed in the frequency-modulation mode with a qPlus sensor with a resonance frequency of 29 to 33 kHz and a stiffness of *k* = 1.8 kN/m. Typical image parameters were an amplitude *A* = 500 pm and a frequency-shift set point of Δ*f* = +10 to +25 Hz. Data and image processing was performed with MATLAB (The MathWorks, Inc.) and WSxM [28].

## Results and Discussion

### Initial experiments with poking holes

**Figure 2:**
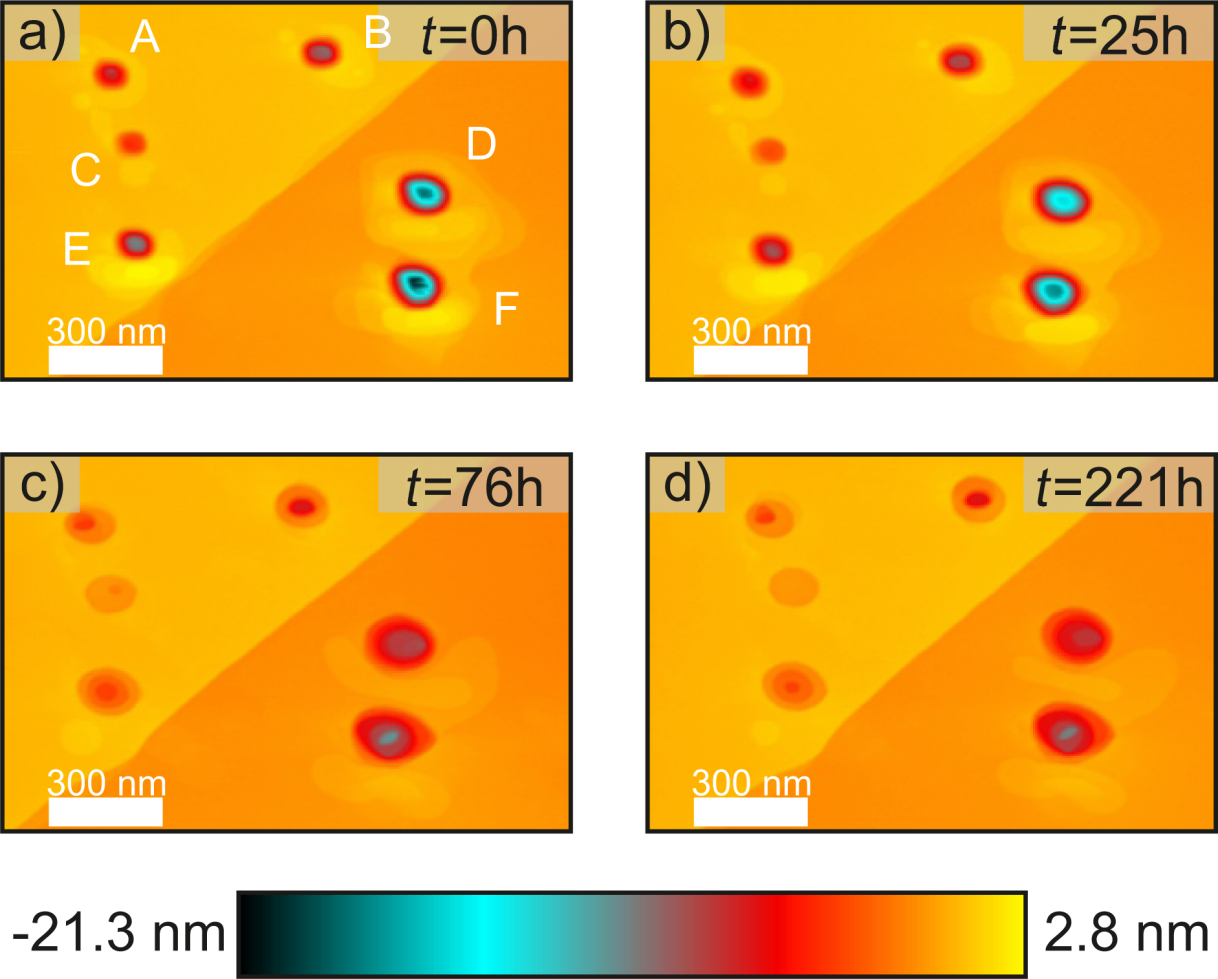
1.5 μm × 1.0μm sections showing the time evolution of artificial defects and accumulations in three ranges of relative humidity. (a to b) 1st range: 20.5% *<* RH *<* 25.0%, *t* = 0–27 h); (b to c) 2nd range: 25.0% *<* RH *<* 35.1%, *t* = 27–73 h; (c to d) 3rd range: 18.5% *<* RH *<* 23.0%, image (c) was recorded directly after decreasing the humidity at *t* = 76 h). In image (a) the holes are denominated from A to F for depth comparison below in Figure 3. Measurement parameters: *f*_0_ = 32893 Hz, *A* = 500 pm, Δ*f* = 20 Hz.

This experiment shall investigate qualitatively how the material transport rate changes with humidity RH in a range of 18.8% *<* RH *<* 35.1%. Several holes and accumulations of different size and shape were induced by poking the tip several nanometers into the surface. The defects initially have a conical shape with depths ranging from 1 to 18 nm and diameters between 100 and 200 nm. The depth of the defects is defined by the height difference between the lowest point of the defect and the surrounding terrace. The removed material aggregates around the artificial defect. The accumulations do not attach symmetrically but irregularly around the holes. This can be explained by the shape and angular orientation of the tip towards the surface. The consecutive images displayed in Figure 2 are 1.5 μm × 1.0 μm sections of the continuously scanned area and show the time evolution of the shape of the holes.

During this experiment the humidity changed over time. We can devide the experiment into three sections, defined by the relative humidity. The time evolution of the first section, 20.5% *<* RH *<* 25.0% for the first 27 h can be seen by comparing Figure 2a to Figure 2b. The first period shows a slow erosion of the accumulated material and a filling of the defects from the bottom. The accumulated material around both top structures and the middle left structure slightly decreases. For the other three structures a slight spread of the accumulated material can be observed. The lowest levels of the middle and bottom left defects get filled.

In the second section the relative humidity ranges from 25.0% to 35.1% within a time period of 46 h. Figure 2b and Figure 2c show the surface topography directly before and after this period. It can be observed that the accumulated material around the top right and the middle left structure has completely eroded. Also, for the other structures only a fraction of the initial volume is left. All holes appear to be less deep and show an increased diameter. This increase can be understood by considering a simple picture in which the additional energetic cost of a hole can be described by the length of its edge. This model is based on the Terrace Step Kink model as described in Ref. [29]. If we were to start with a defect that has the first layer removed in a circle with radius *R*_1_ and a second (deeper) layer removed in a circle with radius *r*_1_*< R*_1_, this would yield an energy term for the edge *E*_1_ ∝ 2π*R*_1_ + 2π*r*_1_. The defect now evolves, maintaining the total volume of the hole to a single-layer circular defect with radius *R*_2_. Conservation of volume requires


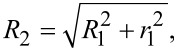


but now the corresponding energy of the edge is smaller,


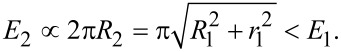


An illustrative drawing can be found in Supporting Information File 1, Figure S5. The material that is filling the smaller hole in the second layer is removed from the edge of the first layer. Therefore the dissolved volume per time *v_t_* is proportional to the edge length 2π*R*_1_ with *V_t_* = 2π*R*_1_*c_Vt_*, where [*c_Vt_*] = m^2^/s is the material transport coefficient. This results in a depth change *d*Δ*z*/d*t*:

[3]
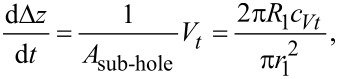


with *A*_sub−hole_ being the area of the layer being filled. The 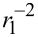 relation shows why small and steep holes fill up faster than larger and shallow structures. For steep accumulations this results in a spreading of the material from the higher levels into the surrounding area, which can be observed in the experiment described in the next section.

**Figure 3:**
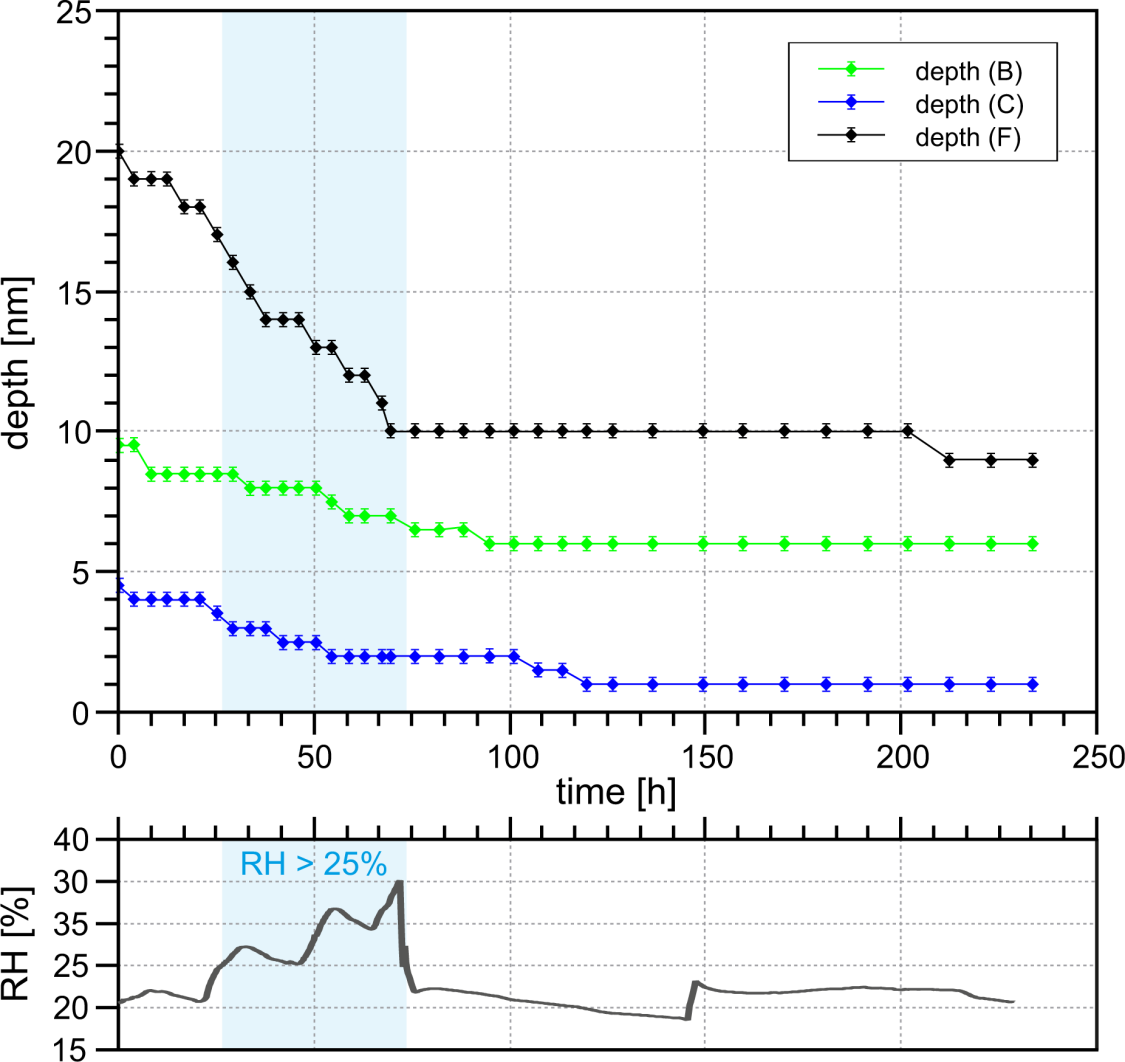
Maximum depth of the observed holes over time. The poking holes to which the letters correspond is displayed in Figure 2 a). The blue highlighted area shows the second time period with RH > 25%. Only holes (B,C,F) are displayed, the full graph is displayed in Supporting Information File 1.

In the third section the relative humidity ranges from 18.5% *<* RH *<* 23.0%. Figure 2c and Figure 2d show the time evolution within the next 145 h. The amount of accumulated material of the bottom right and the middle right structure has further decreased, yet less significant compared to the previous section. Only the lowest level of the middle and bottom holes have partially filled. The time evolution of the maximum depth is displayed in Figure 3. During the first period the steep holes (especially D and F) show significant a decrease in depth that continues (see D and F) or is enhanced (see E) for the second period. The third section only shows small changes.

This experiment indicates that the material transport for steep structures is significantly enhanced driven by the energetically unfavorable form (large surface-to-volume ratio). A contribution of the relative humidity cannot be derived readily from this experiment. The tip–sample-convolution effect described above appears to be a dominant factor. For that reason, further experiments were performed by scratching holes into the substrate.

### Scratching holes at RH < 6%

In this experiment the surface was exposed to very dry air below RH *<* 6%. Therefore, a low coverage of the surface with water molecules is expected. When probing the surface at such low humidity, the mobility of ions is expected to be reduced.

**Figure 4:**
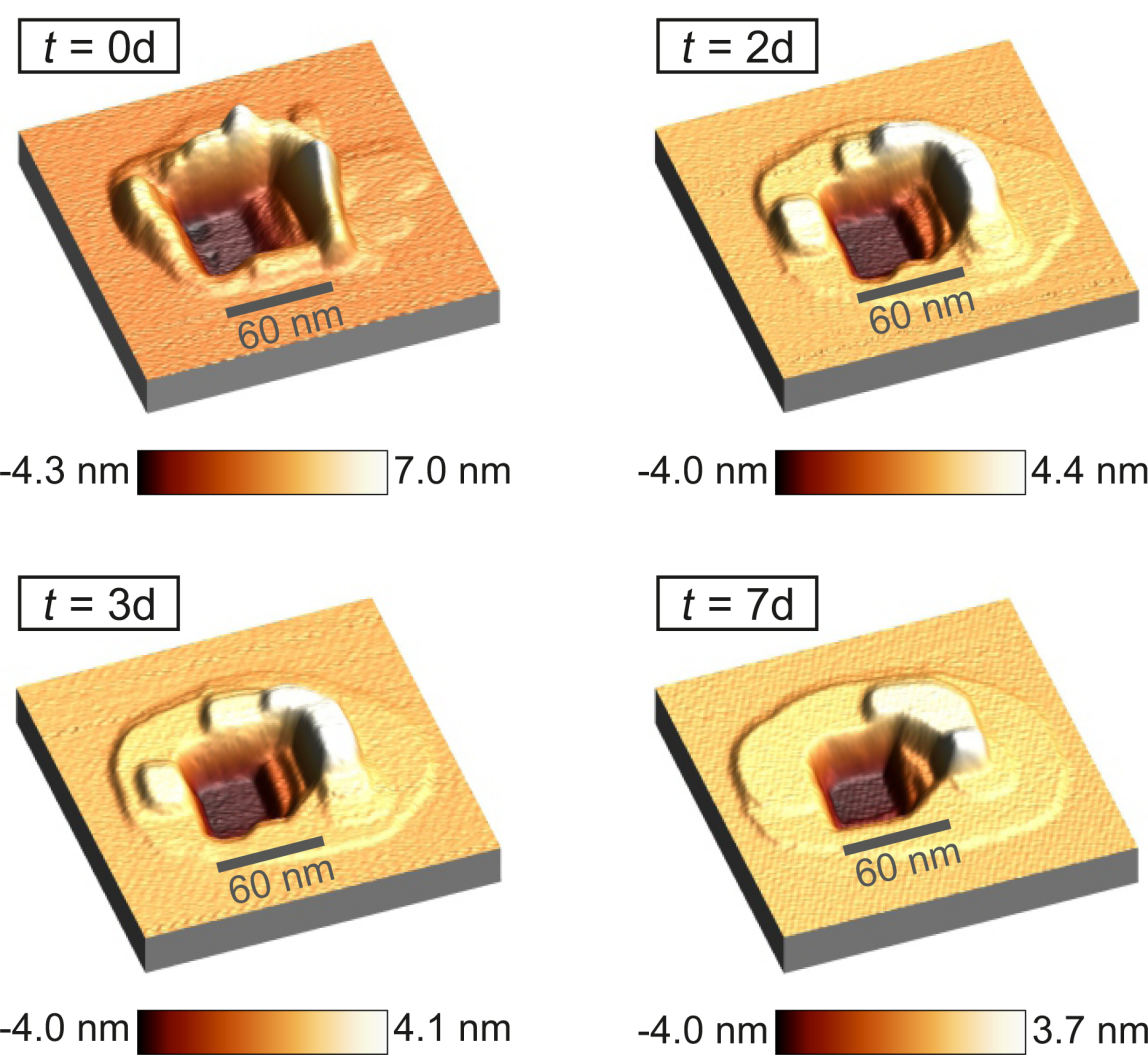
200 nm × 200 nm 3D profile image of the generated scratching site directly taken after generating, after two, three and seven days at 3.0 < RH < 5.5%. The lowest levels of the hole fill until the second measurement, yet the maximum depth remains constant afterwards. The higher levels accumulated material erodes more quickly. A spread of the material around the defect can be observed. Measurement parameters: *f*_0_ = 31785 Hz, *A* = 500 pm, Δ*f* = 25 Hz.

A large rectangular defect with an edge length of about 80 nm × 60 nm with a depth of up to 4.3 nm (see Figure 4) was created with the scratching technique described above. The removed material accumulated in several nanometer thick and up to 7.0 nm high walls around the hole. The area was scanned only once every few days to minimize the effects of tip-induced material transport. In the seven days of observation the relative humidity was kept between 3.0% ≤ RH ≤ 5.5%.

In order to evaluate the size of the accumulation and the defect every measurement point that differs more than 165 pm (half an atomic step height) from the average height of the surrounding plain (*z* = 0) is referred as part of the hole or the accumulation. The volume is obtained by adding up the height of all corresponding measurement points multiplied with the size per point. The total volume of the hole or accumulation therefore can be expressed by

[4]
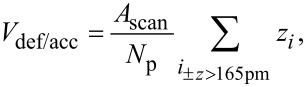


where *A*_scan_ is the size of the total scan area and *N*_p_ is the number of measurement points.

Over a period of seven days significant changes of the studied structure can be observed. The accumulation erodes quite fast at the beginning and spreads out to the surrounding area. Initially, the defect was surrounded by walls which were up to 35 nm thick and up to 7 nm high. After two days the maximum height decreased to 4 nm and a single atom layer is spreading around the structure. At the defect a rounding of the corners can be observed. These processes continue over time.

**Figure 5:**
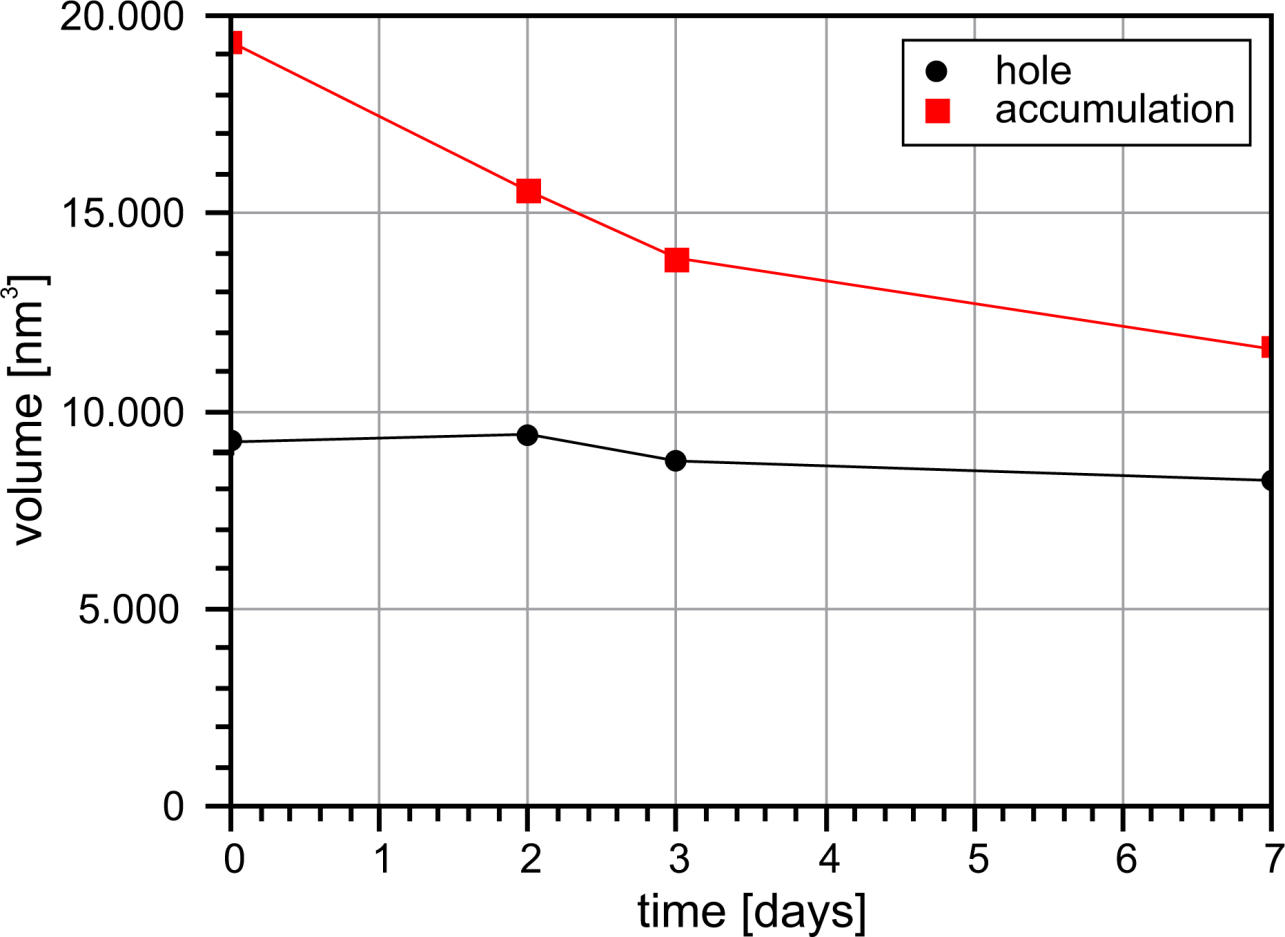
Volume of the hole and accumulation at 3.0% *<* RH *<* 5.5% within seven days. No significant change in the volume of the hole can be observed in this time period. The accumulation appears to be larger and show a higher material transport than the hole. However, these values are influenced by the tip–sample convolution.

Figure 5 shows the size and change rate for each image. It can be seen that the change of the material accumulation is much larger than that of the defect. This would mean a loss of material as the total change rates do not match. This can be due to the amplification/reduction effect of steep convex/concave structures as described above. Furthermore, this scan area is not a closed system and allows for material exchange with the surroundings. Also material gets removed by the tip. However, there is one important observation to be made: Slow material movement still takes place despite the very low humidity. The observed slower filling of the defect compared to the erosion of the accumulated material could be either a measurement artifact (assisted mobility) or a real physical effect. While in this example it is difficult to differentiate, further examples shown in this work all show a similar pattern that the accumulated material erodes quicker than the defect fills. This makes us conclude that it is indeed a real physical effect.

### Scratching holes at 12% < RH < 20%

**Figure 6:**
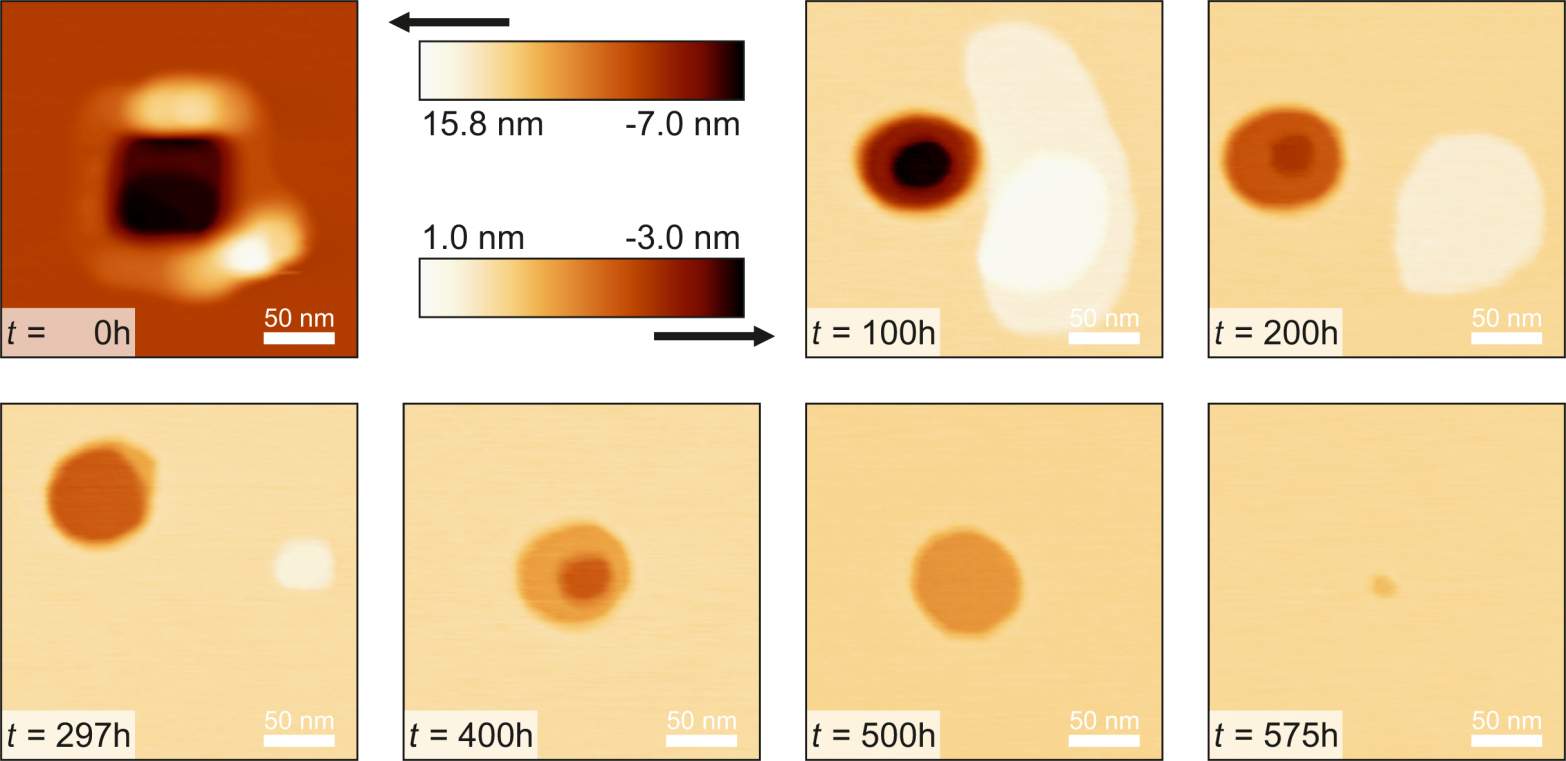
250 nm × 250 nm images of the time evolution of both defect and accumulation at different consecutive times. It can be observed that both hole and accumulation turned in a round shape. For better visibility the first image at *t* = 0 shows a different color scale.

**Figure 7:**
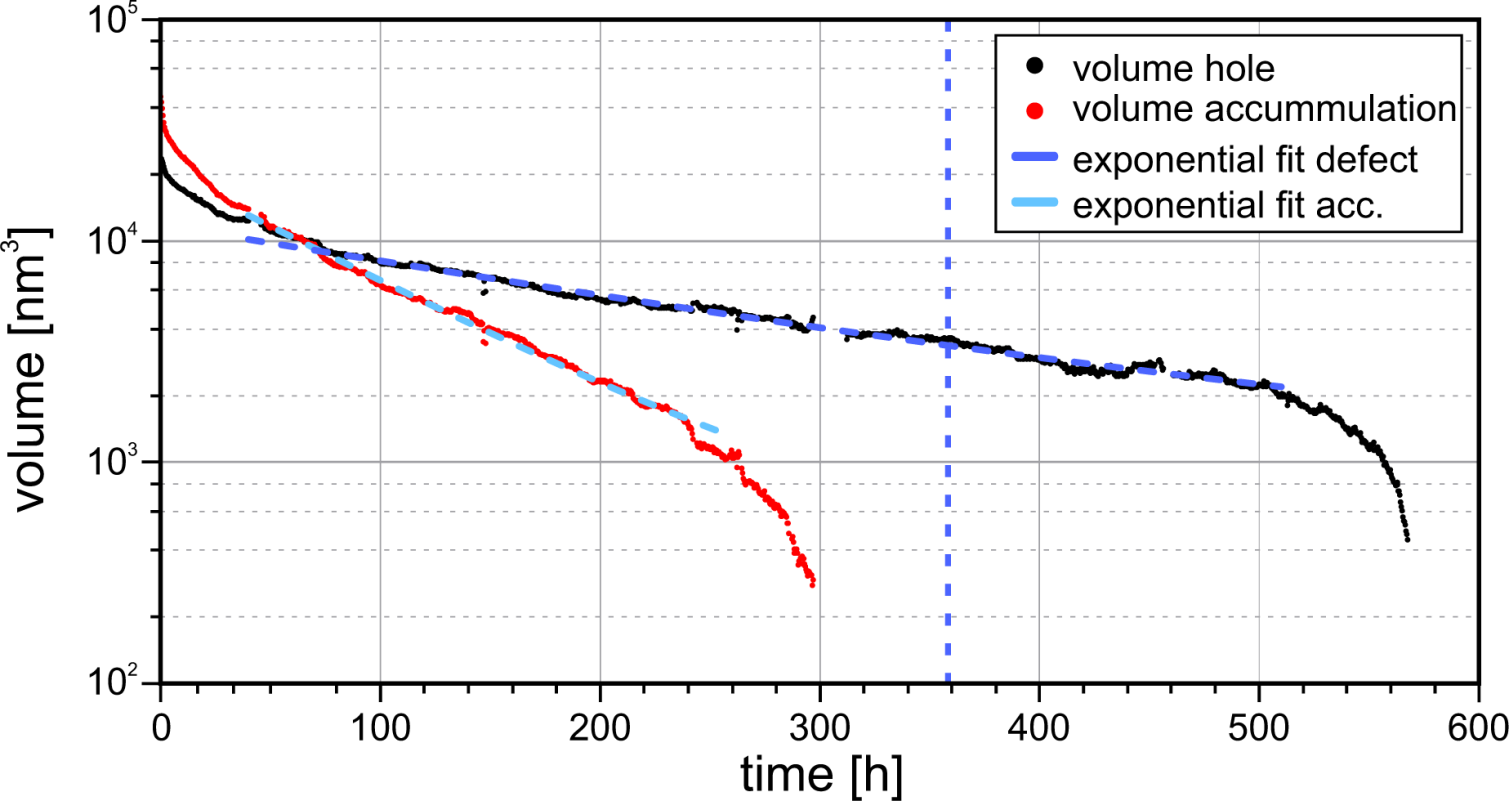
Time evolution of both defect and accumulation volume. After an initial settling time the logarithmically scaled size values show a linear behavior, which corresponds to an exponential decrease (see solution of differential Equation 5). The dashed blue vertical line corresponds to the point after which the relative humidity was increased linearly from RH = 12.9% to 19.9% until complete filling. The exponential behavior is not affected. When the structure volumes fall below a certain size (accumulation: *t >* 250 h, hole: *t >* 510 h) the transport rate increases and now corresponds to Eqaution Equation 6. Measurement parameters: *f*_0_ = 29302 Hz, *A* = 500 pm, Δ*f* = 10 Hz.

In this experiment material transport was investigated at relative humidities from 12% ≤ RH ≤ 16% for 15 days and then it was linearly increased by about ΔRH = 1% per 19 h to investigate whether this affects the transport rate. The temperature was 20 ± 1 °C. A rectangular defect with edge lengths of about 95 nm × 80 nm and a maximum depth of 7.0 nm was created. The accumulation has an apparent initially volume of approximately 136000 nm^2^ and the defect has an approximate volume of 72000 nm^2^. The time evolution of the structure is shown in Figure 6 and Figure 7 (graph). It can be seen that after an initial settling time the accumulation, as well as the defect, get exponentially smaller, until the size drops below a certain value.

The phenomenon of an enhanced material transport directly after creating a structure can be explained by the sample material being ripped out of the crystal structure during the scratching process. Now, the material is likely no longer in a monocrystalline configuration like the bulk material. The material accumulation therefore is less stable and more mobile. This hypothesis explains the increased volume change during the first hours compared to the exponential decrease afterwards. Conversely, this results in an increased availability of material and a quicker filling of the hole.

The accumulated material erodes faster than the hole is filled. The accumulation completely eroded after about 12.5 days. At this time the volume of the hole has shrunk to 17% of its initial volume. About 2.5 days later we started to increase the relative humidity, marked by the blue dashed vertical line. The exponential decrease of the hole did not significantly change with the increase of humidity, but rather when it fell below a certain size at approximately *t* = 510 h, when the humidity reached RH = 17.9%. When the hole completely filled, the relative humidity had reached 19.9%.

For a quantitative description of this behavior we consider the sample material to be able to take two different states: (1) In the bound state the K^+^/Br^−^-Ions are immobilized on the surface. This state is observed by the AFM technique as the surface topography. (2) In the second state the ions are dissolved in the hydration layer or in a physiosorbed or precursor state [30], which shows a high mobility and cannot be imaged. The material from the accumulation over time changes from the first state into the second state and from the second state into the first state, attaching to the hole. These transitions require each atom to overcome an energy barrier *E*_b_. The change rate can be expressed by

[5]
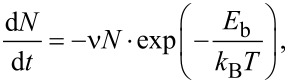


with *N* being the size of the observed structure, ν the attempt frequency to overcome the energy barrier, *k*_B_ the Boltzmann constant and *T* the temperature. We assume an attempt rate of ν = 10^13^ s^−1^, which is in the order of magnitude of a KBr phonon [31], equal for both transitions. This differential equation can be solved by an exponential decaying function of the form *N*(*t*) = *N*_0_·exp(−*t*/τ), with *N*_0_ being the structure size at the time *t* = 0 (in this case the time after the initial enhanced transport) and 1/τ the exponential decay rate. This allows us to calculate a value for the energy barrier from the recorded data, given by

[7]
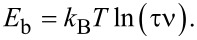


As we consider the exponential decay rate to be constant we obtain a value for the energy barrier of *E*_b,acc_ = 875 meV for the accumulation and *E*_b,def_ = 895 meV for the defect. The calculated values vary by less than 6.8% when ν is altered by one order of magnitude. If we calculate the barrier for the defect before and after the point of increasing the relative humidity separately our results differ by less than 2.6%.

To explain the non-exponential behavior as the accumulation and defect get smaller, a different model is required. In the model described above the ions dissolve or adsorb at any position of the interface of the structures, which then rearranges into the energetically more favorable round shape. For smaller sizes a further effect becomes dominant. As the step edges make up the highest energy cost of the surface configuration they are more reactive than the rest of the surface. The ions face a lower energy barrier and if the ratio between edge length and structure size is large enough the material transport is mainly driven by adsorption and dissolution at the step edges. To understand why that would lead to higher material transport, we consider a circular accumulation or defect with a total number of atoms *N*. With a radius *r* and interatomic spacing of *a*:

[8]
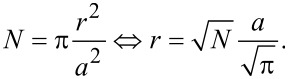


The number of atoms at the step edge *N*_e_ can be described by

[9]
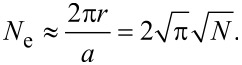


If material transport is confined to the edges, then (see Equation 5):

[10]
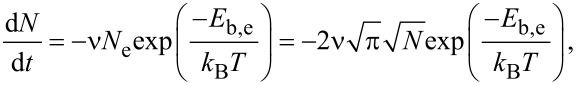


with *E*_b,e_ being the energy barrier height at the step edge. This yields a solution of the form

[6]
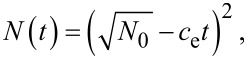


with 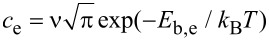, and explains the increased decay of the structure volumes as their size decreases.

Further measurements at a relative humidity in a similar range of 14.5% *<* RH *<* 18.5% are shown in Figure S6 in Supporting Information File 1. Several structures of different volumes were observed and show a similar behavior. The accumulated material erodes faster than the holes fill. Slow material transport (compared to the experiment at RH = 28%, next section) is taking place. The smallest structure fills within less than 200 h whereas no significant change in volume for the largest structures can be observed. These measurements are not included to this manuscript as they were created by poking but not with scratching. The measurement of the exact volumes therefore may be imprecise.

### Scratching a hole at RH = 28%

In this experiment a scratching defect at *RH=* 28.1% and *T* = 22 °C was observed. A coverage between one and three molecular water layers is expected [1,4]. A quick first image was taken immediately after creation of the defect (see Supporting Information File 1, Figure S1). Already in this first image, the hole does not appear as a square, as it did in Figure 4, but rather it is already rounded.

**Figure 8:**
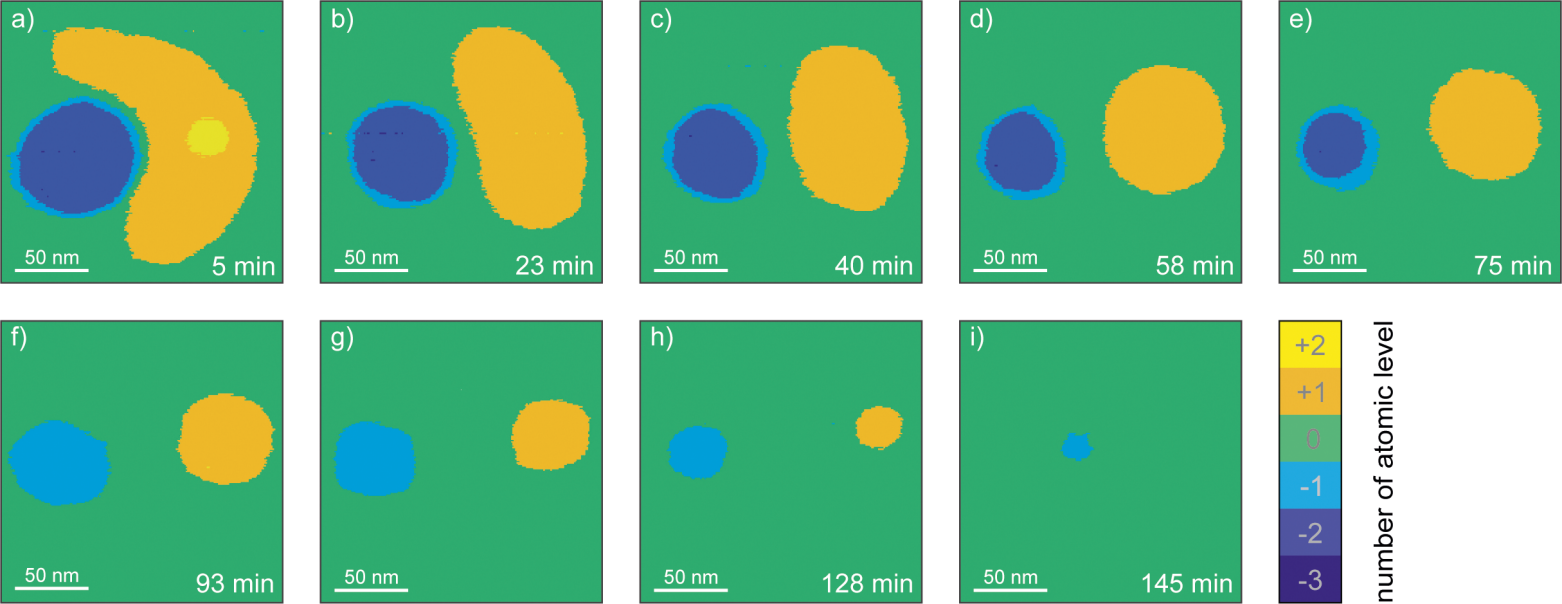
200 nm × 200 nm sections of the time evolution of the scratching site at RH = 28.2%. Each image is measured within 17.5 min starting about 5 min after the creation. The initial rectangular scratching site adopts in a few minutes a circular form. Similarly the material accumulation erodes faster on the sharp margins and also adopts a circular form. The small second layer erodes after some minutes. The hole, already after a few minutes is largely two atomic layers deep. The accumulation completely erodes some minutes faster than the deepening. Measurement parameters: *f*_0_ = 32873 Hz, *A* = 500 pm, Δ*f* = 20 Hz.

The sample was then continuously scanned with a period of *t* = 1050 s = 17.5 min per frame, shown in Figure 8. Each measurement point was assigned to a certain atom layer and represents an area of 1 nm^2^. For a quantitative analysis of the material movement, the topographic images of this experiment were converted from the continuous height scale into a discrete scale.

**Figure 9:**
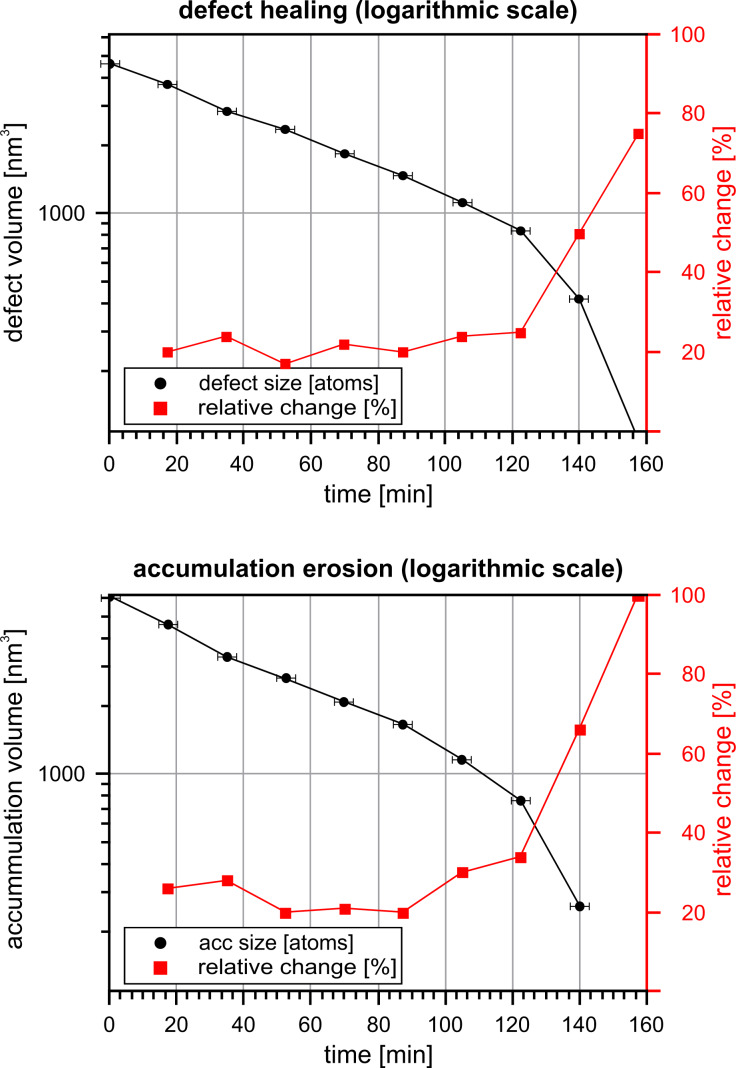
Time course of the size and change rate of the observed defect (upper position) and accumulation (lower position) at RH = 28.2%. For larger structures the total size shows an (approximately) linear decrease on a logarithmic scale. The relative change describes the ratio between the transported material and the previous structure volume per each timestep of 1050 s. For larger structures this value stays roughly constant.

The volume of the defect and the accumulation are shown as a function of time in Figure 9 and in Table S1 in Supporting Information File 1. Again, there is an exponential relationship until approx. minute 100. Regarding this time period, we applied the same analysis as we did earlier, and obtained the values for the transition energy barrier of *E*_b,acc_ = 867 meV for the accumulation and *E*_b,def_ = 874 meV for the hole.

The experiment at a relative humidity of 14.5% *<* RH *<* 18.5% in Figure S6 in Supporting Information File 1 (described in the previous section) show structures of different volumes, larger as well as smaller than the defect in this experiment. However, even the smallest hole (smaller than here) is filled in a time period that is one to two orders of magnitude longer. This observation supports the interpretation that in this experiment the material transport is dominantly affected by the relative humidity and is significantly increased compared to values below RH *<* 20%.

## Conclusion

Our studies of defect healing and erosion of accumulations in various ranges of relative humidity have shown that the speed of the material movement depends on several factors including the relative humidity, and size and shape of the accumulation or defect. Directly after the scratching process the material that is ripped out of the crystal structure does not fully realign. Therefore, it is less stable and more mobile or more likely to dissolve in the water film. This leads to a short time period where the transport rate is strongly enhanced. The relative humidity is one of the most important factors, but it has a non-linear effect on material transport. It has been shown that material transport is still possible at very low relative humidities. It also has been observed that artificially generated material accumulations are less stable than holes. Furthermore, small structures show a large surface compared to the volume and are, therefore, energetically less favorable. This results in the healing of small defects within a few hours to a few days, whereas larger defect may remain without significant changes over a period of some weeks.

## Supporting Information

File 1 Additional experimental data.

## References

[1] Asay D B, Kim S H (2005). J Phys Chem B.

[2] Ewing G E (2006). Chem Rev.

[3] Luna M, Rieutord F, Melman N A, Dai Q, Salmeron M (1998). J Phys Chem A.

[4] Arima K, Jiang P, Deng X, Bluhm H, Salmeron M (2010). J Phys Chem C.

[5] Hu J, Xiao X-D, Ogletree D F, Salmeron M (1995). Science.

[6] Miranda P B, Xu L, Shen Y R, Salmeron M (1998). Phys Rev Lett.

[7] Wastl D S, Weymouth A J, Giessibl F J (2013). Phys Rev B.

[8] Pürckhauer K, Kirpal D, Weymouth A J, Giessibl F J (2019). ACS Appl Nano Mater.

[9] Giessibl F J (1998). Device for contactless scanning of surface. DE.

[10] Giessibl F J (1998). Appl Phys Lett.

[11] Giessibl F J (2019). Rev Sci Instrum.

[12] Gross L, Mohn F, Moll N, Liljeroth P, Meyer G (2009). Science.

[13] Huber F, Matencio S, Weymouth A J, Ocal C, Barrena E, Giessibl F J (2015). Phys Rev Lett.

[14] Pürckhauer K, Weymouth A J, Pfeffer K, Kullmann L, Mulvihill E, Krahn M P, Müller D J, Giessibl F J (2018). Sci Rep.

[15] Ichii T, Negami M, Sugimura H (2014). J Phys Chem C.

[16] Ooe H, Kirpal D, Wastl D S, Weymouth A J, Arai T, Giessibl F J (2016). Appl Phys Lett.

[17] Burnham N A, Colton R J (1989). J Vac Sci Technol, A.

[18] Giessibl F J (1997). Phys Rev B.

[19] Giessibl F J, Bielefeldt H, Hembacher S, Mannhart J (1999). Appl Surf Sci.

[20] Gorb E V, Hosoda N, Miksch C, Gorb S N (2010). J R Soc, Interface.

[21] Pishchik V, Lytvynov L A, Dobrovinskaya E R (2009). Sapphire.

[22] Wei Z, Zhao Y-P (2007). J Phys D: Appl Phys.

[23] Wastl D S, Weymouth A J, Giessibl F J (2014). ACS Nano.

[24] Haynes W M (2015). CRC Handbook of Chemistry and Physics.

[25] Giessibl F J, Binnig G (1992). Ultramicroscopy.

[26] Odin C, Aimé J P, El Kaakour Z, Bouhacina T (1994). Surf Sci.

[27] Winzer A T, Kraft C, Bhushan S, Stepanenko V, Tessmer I (2012). Ultramicroscopy.

[28] Horcas I, Fernández R, Gómez-Rodríguez J M, Colchero J, Gómez-Herrero J, Baro A M (2007). Rev Sci Instrum.

[29] Oura K, Katayama M, Zotov A V (2003). Surface Science.

[30] Brown D E, Moffatt D J, Wolkow R A (1998). Science.

[31] Safron S A, Duan J, Bishop G G, Gillman E S, Skofronick J G (1993). J Phys Chem.

