# Supplementary material for: Ion mobility and material transport on KBr in air as a function of the relative humidity
Source: Beilstein J Nanotechnol. 2019 Oct 30;10:2084–93. doi: 10.3762/bjnano.10.203 (PMC6839551; doi:10.3762/bjnano.10.203)
Supplement: File 1 — Additional experimental data. [file Beilstein_J_Nanotechnol-10-2084-s001.pdf]

BEILSTEIN JOURNAL OF NANOTECHNOLOGY

Supporting Information

for

Ion mobility and material transport on KBr in air as a function of the relative humidity

Dominik J. Kirpal, Korbinian Pürckhauer, Alfred J. Weymouth and Franz J. Giessibl

Beilstein J. Nanotechnol. **2019,** *10,* 2084–2093. doi:10.3762/bjnano.10.203

Additional experimental data

License and Terms: This is a supporting information file under the terms of the Creative Commons Attribution License (http://creativecommons.org/licenses/by/4.0). Please note that the reuse, redistribution and reproduction in particular requires that the authors and source are credited.
The license is subject to the *Beilstein Journal of Nanotechnology* terms and conditions: (https://www.beilstein-journals.org/bjnano)

Supporting Information

for

Ion mobility and material transport on KBr in air as a function of the relative humidity

Dominik J. Kirpal*[1], Korbinian Pürckhauer[1], Alfred J. Weymouth[1] and Franz J. Giessibl[1]

Address: [1]Institute of Experimental and Applied Physics, University of Regensburg, 93040 Regensburg, Germany

Email: Dominik J. Kirpal - dominik.kirpal@ur.de

* Corresponding author

Additional experimental data

Material transport at RH = 28%

Table S1 shows the values corresponding to Figure 7 in the main article. Figure S1 displays the fast scanned image preceding the measurements displayed in Figure 6 in the main article.

Table S1: Temporal evolution of the size and change of the defect and accumulation for the experiment at RH = 28.2%. The accumulation decays after about 150 min, the defect after about 170 min. For larger structures the percentage change fluctuates slightly, around 17–25% per scan frame for the defect and around 20–30% for the accumulation. This corresponds to an approximately exponential change.

frame	time [min]	number of atoms defect	change total	change percentage	number of atoms accumulation	percentage total	change change
0	4	128373			170968		
1	22	103330	−25043	−20%	127189	−43779	−26%
2	39	78618	−24712	−24%	91442	−35747	−28%
3	57	65178	−13440	−17%	73100	−18342	−20%
4	74	50977	−14201	−22%	57742	−15358	−21%
5	92	40677	−10300	−20%	45937	−11805	−20%
6	109	31028	−9649	−24%	32020	−13917	−30%
7	127	23253	−7775	−25%	20976	−11044	−34%
8	144	11521	−11732	−50%	7142	−13834	−66%
9	162	2846	−8675	−75%	0	−7142	−100%

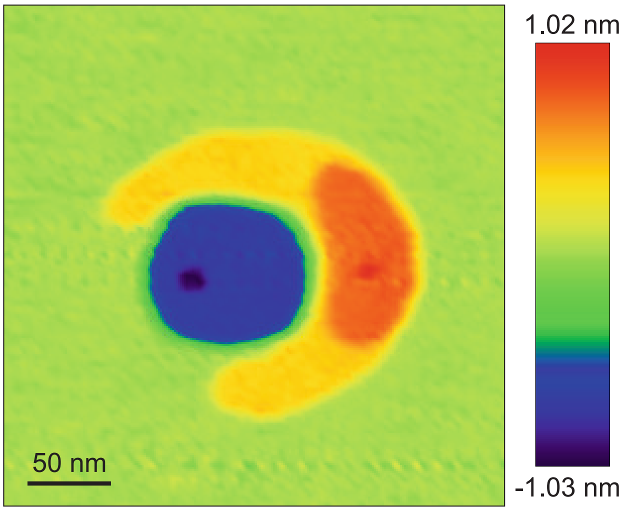

Figure S1: Fast scanned first image (256 s) at RH = 28%.

Comparison of RH < 25% and RH > 25%

Figure S2 and Figure S3 display intermediate steps of the 221 h time evolution at different relative humidities displayed in Figure 2 in the main article. Figure S4 is the full graph corresponding to Figure 3 in the main article.

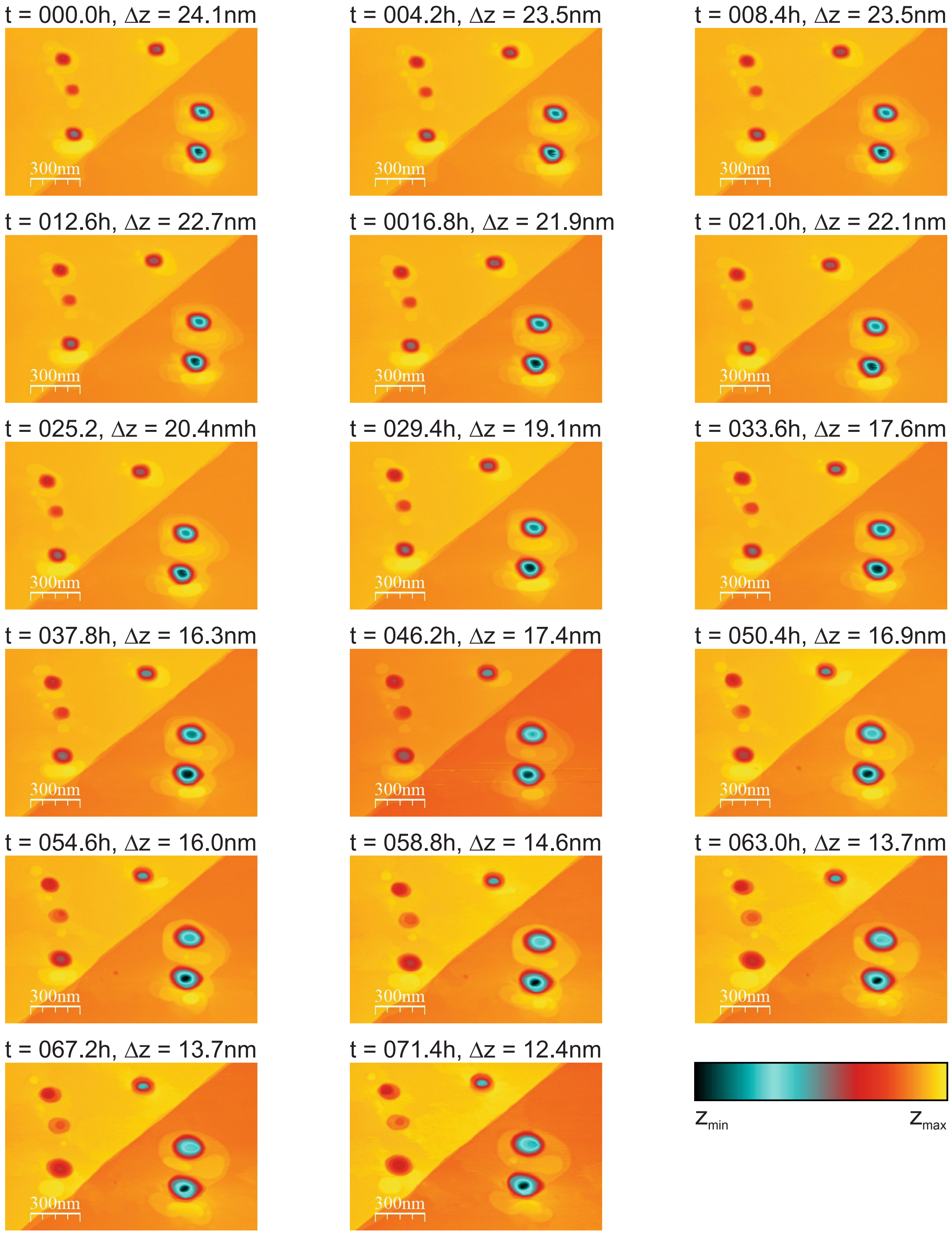

Figure S2: 1.5 µm × 1.0µm, f_0 = 32893 Hz, A = 500 pm, Δf = 20 Hz, t = 0 h to 27 h), 20.5% < RH < 25.0%, t = 27 h to 72 h, 25.0% < RH < 35.1%, t > 72 h, 18.5% < RH < 23.0% (part 1/2)

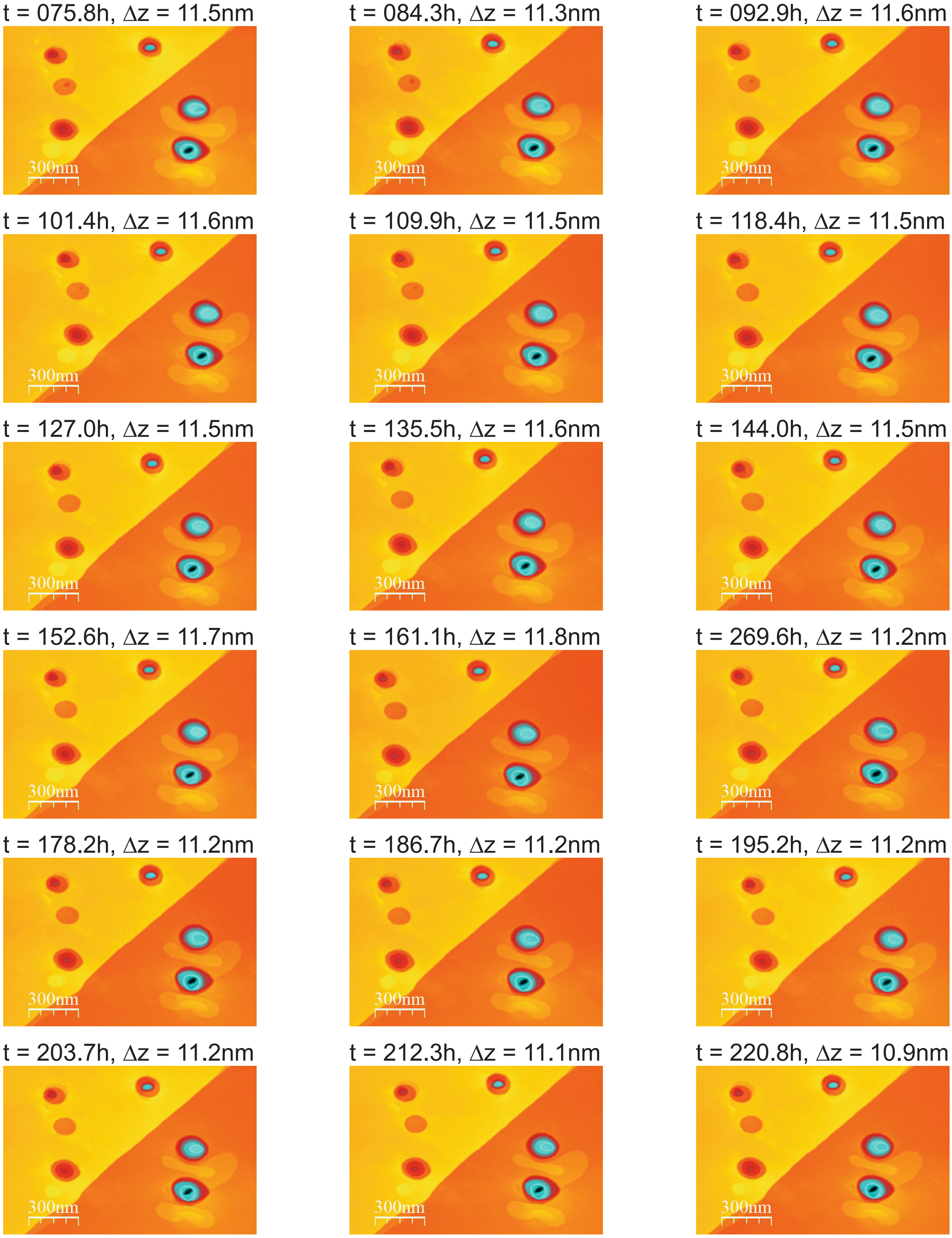

Figure S3: 1.5 µm × 1.0µm, f_0 = 32893 Hz, A = 500 pm, Δf = 20 Hz, t = 0 h to 27 h), 20.5% < RH < 25.0%, t = 27 h to 72 h, 25.0% < RH < 35.1%, t > 72 h, 18.5% < RH < 23.0% (part 2/2)

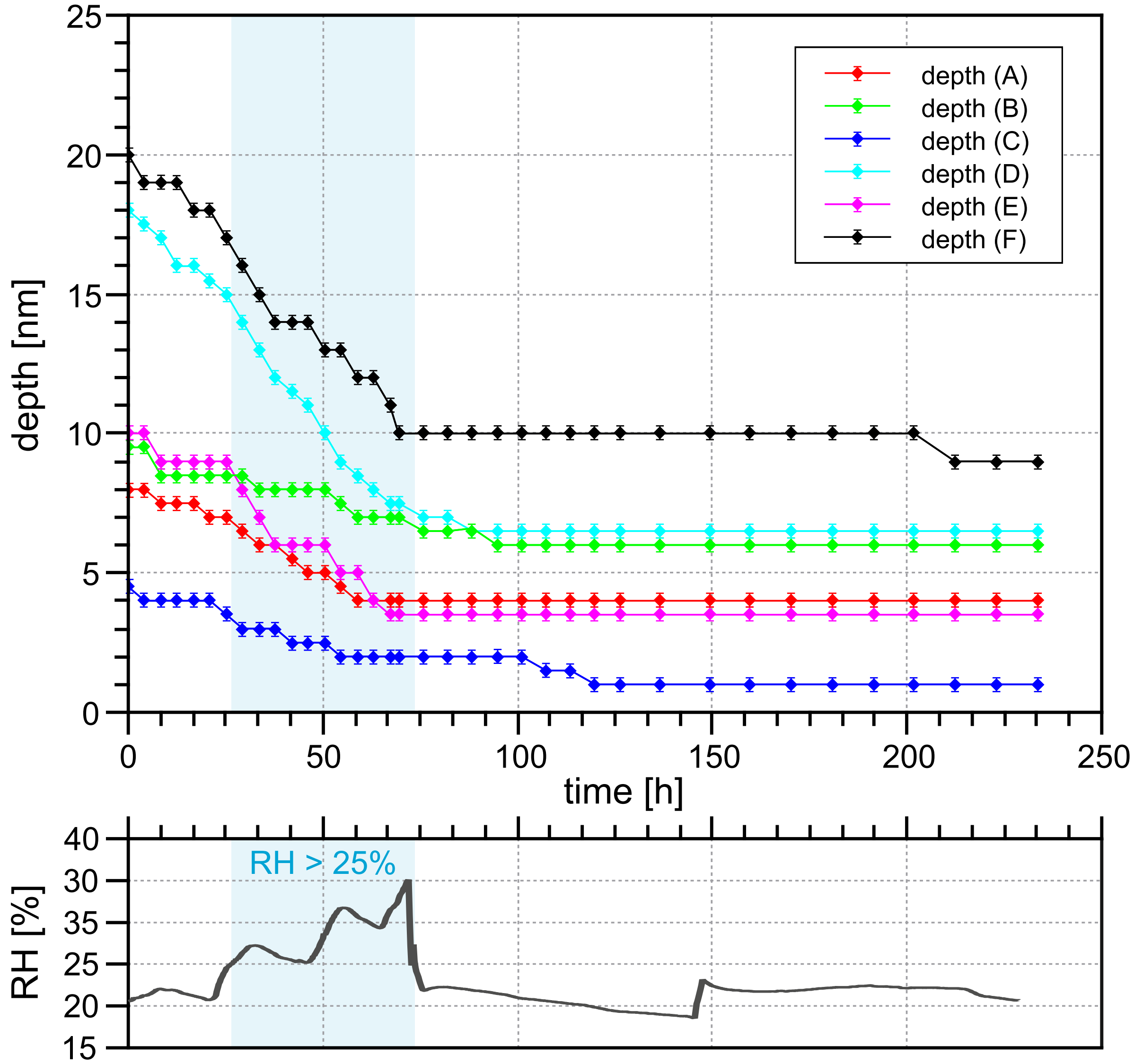

Figure S4: Full graph of the maximum depth of the observed holes over time. The features corresponding to each letter A to F are displayed in Figure 2a in the main article. The blue area highlighted in blue shows the second time period with RH > 25%.

Increase of the diameter of poking holes

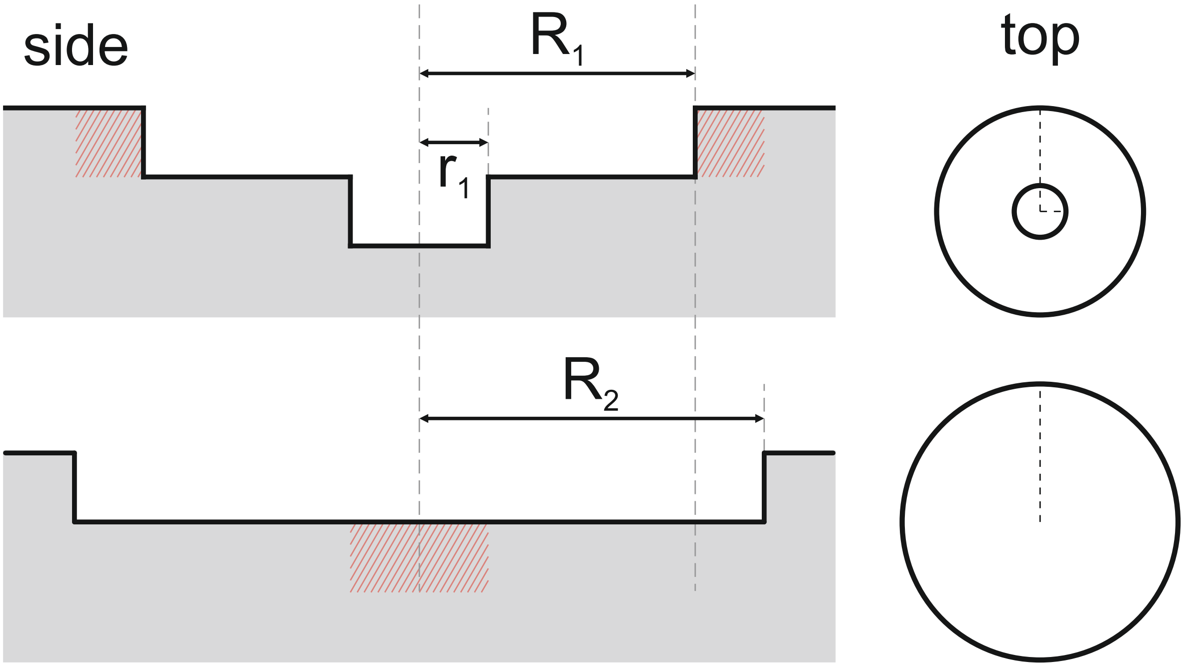

Figure S5: Schematic drawing of two defects with same volumes but different shape. The first defect shows two layers, whereas the second only one with an increased radius $R_2 = \sqrt{r_1 + R_1}$. For the same volume the edge length of both layers in the first holes is bigger than that of the second hole. The surface energy of the second structure therefore can be considered to be smaller $E_2 \propto \pi R_2 = \pi\sqrt{R_1 + r_1} < E_1 \propto \pi r_1 + \pi R_1$.

Additional data for 14.5% < RH < 18.5%

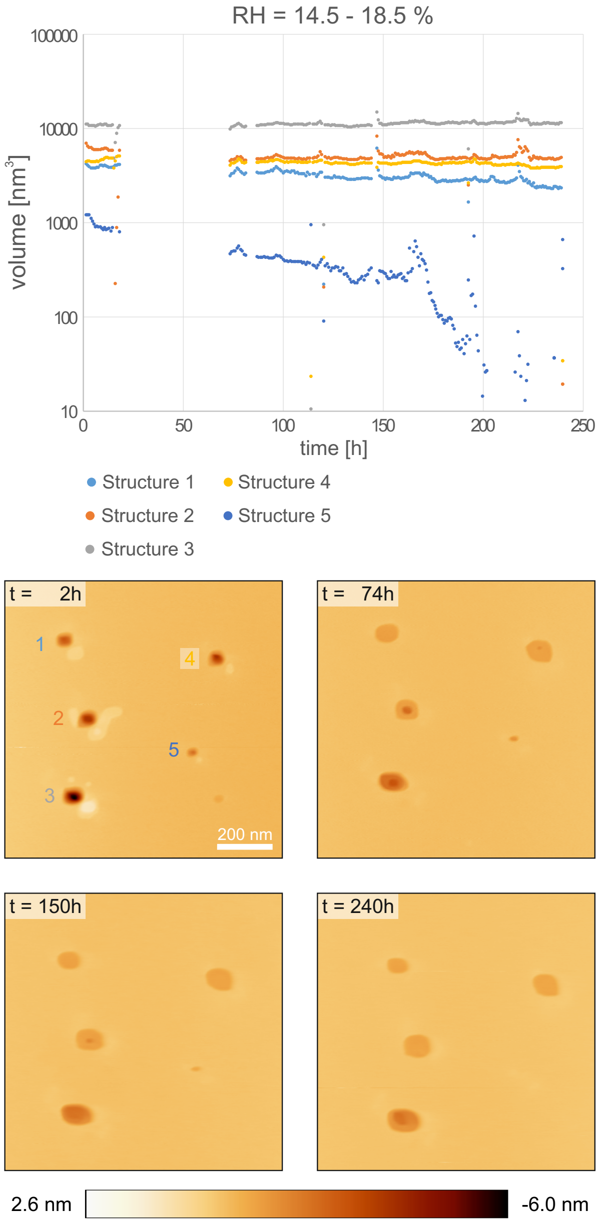

Figure S6: Additional data at 14.5% < RH < 18.5% and structure sizes comparable to the experiments performed at 12% to 16% and 28.2%. The material transport at this relative humidity is significantly slower, compared to RH = 28%, despite having similar volumes.
